# Polygenic risk scores and breast cancer risk prediction

**DOI:** 10.1016/j.breast.2023.01.003

**Published:** 2023-01-10

**Authors:** Eleanor Roberts, Sacha Howell, D Gareth Evans

**Affiliations:** a Division of Cancer Sciences, Faculty of Biology, Medicine and Health, University of Manchester, Manchester Academic Health Science Centre, Manchester, UK; b Manchester Centre for Genomic Medicine, Manchester University Hospitals NHS Foundation Trust, Manchester, UK; c Division of Evolution, Infection and Genomics, School of Biological Sciences, Faculty of Biology, Medicine and Health, University of Manchester, Manchester Academic Health Science Centre, Manchester, UK; d Nightingale/Prevent Breast Cancer Centre, Wythenshawe Hospital, Manchester University NHS Foundation Trust, Manchester, UK; e Manchester Breast Centre, Manchester Cancer Research Centre, The Christie Hospital, Manchester, UK

**Keywords:** Polygenic, Risk, Scores, Breast, Cancer, Prediction, screening, Prevention

## Abstract

Polygenic Risk Scores (PRS) are a major component of accurate breast cancer risk prediction and have the potential to improve screening and prevention strategies. PRS combine the risk from Single nucleotide polymorphisms (SNPs) associated with breast cancer in Genome Wide Association Studies (GWAS) and explain over 30% of breast cancer heritability. When incorporated into risk models, the more personalised risk assessment derived from PRS, help identify women at higher risk of breast cancer development and enables the implementation of stratified screening and prevention approaches. This review describes the role of PRS in breast cancer risk prediction including the development of PRS and their clinical application. We have also examined the role of PRS within more well-established risk prediction models which incorporate known classic risk factors and discuss the interaction of PRS with these factors and their capacity to predict breast cancer subtypes. Before PRS can be implemented on a population-wide scale, there are several challenges that must be addressed. Perhaps the most pressing of these is the use of PRS in women of non-White European origin, where PRS have been shown to have attenuated risk prediction both in discrimination and calibration. We discuss progress in developing and applying PRS in non-white European populations. PRS represent a significant advance in breast cancer risk prediction and their further development will undoubtedly enhance personalisation.

## 1 Introduction: prognosis and diagnosis

Breast cancer is the world's most prevalent cancer with 7.8 million women alive who have been diagnosed with breast cancer between 2015 and 2020. In 2020, there were 2.3 million women diagnosed globally with 685,000 of these women dying from the disease [1]. Breast cancer occurs in every country of the world and can affect any woman who is post-pubertal with prevalence increasing with age. The earlier breast cancer is detected, the greater the chances of long-term survival.

**Fig. 1:**
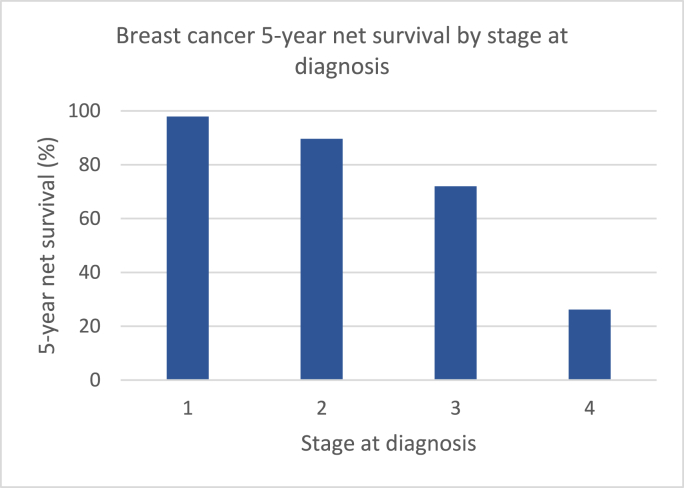
5-Year net survival (%) of women diagnosed with BC by the stage at which the BC was diagnosed. All data: adults diagnosed 2013–2017, followed up to 2018. (CRUK 2018.) [2].

Women with breast cancer have much higher chances of survival the earlier the cancer is diagnosed (Fig. 1) and early detection is the key to improving mortality figures. Most developed countries have, therefore, adopted population based mammographic screening programmes in an attempt to reduce tumour stage and improve survival. Although there are small numbers of women, particularly those with a family history and carriers of pathogenic variants in high-risk genes such as *BRCA1, BRCA2* and *TP53,* who benefit from high-risk screening (often including annual MRI), most of the population receives screening based upon age alone. The use of risk models including standard risk factors, such as family history and hormonal/reproductive factors, integrated with mammographic density and DNA testing now offers the opportunity to more accurately discriminate risk to introduce true population-based risk stratification [3].

### 1.1 Genetic predisposition

Breast cancer is a heritable disease with epidemiological studies indicating around 4–5% is due to highly penetrant autosomal dominant predisposition [4]. Twin studies suggest around 27% is likely to have a strong heritable component [5]. There are many high and moderate risk genes that have been associated with BC development and these are detailed in Table 1.

**Table 1:** High and moderate penetrance BC risk genes and the lifetime BC risk that they confer. Adapted from [[6], [7], [8], [9], [10], [11], [12], [13], [14], [15], [16], [17], [18], [19], [20]].

Penetrance	Gene	Lifetime BC Risk (%)	Rate of variant within outbred populations (%)
High	*BRCA1*	55–85	0.12–0.20
High	*BRCA2*	45–69	0.20–0.50
High	*TP53*	56–90	0.02
High	*PTEN*	60	<0.01
High	*STK11*	32–54	<0.01
High	*CDH1*	60	<0.01
Moderate	*ATM*	25	0.2
Moderate	*CHEK2*	40	0.3–0.5
Moderate	*PALB2*	25–40	0.1
Moderate	*BARD1*	20	0.1–0.2
Moderate	*RAD51C*	20	0.1
Moderate	*RAD51D*	20	0.1

**Fig. 2:**
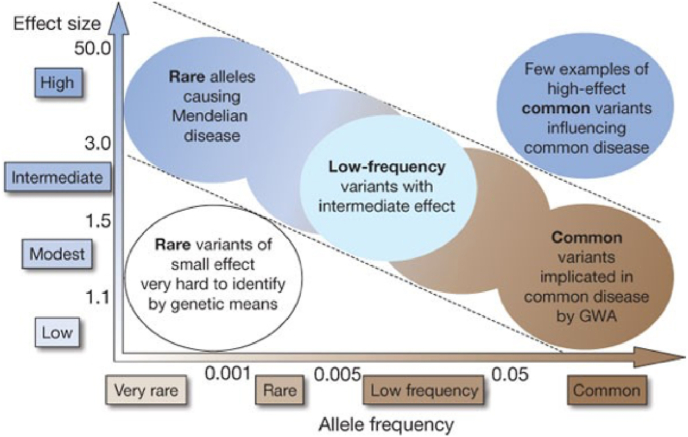
The role of allele variants and the size of the effect they can have on disease penetrance. Generally, rare pathogenic variants have a large impact on disease development and common variants have a smaller impact on disease development [25].

Since the discovery of well-validated high and moderate risk genes, recent research has focused on more common, lower penetrance gene variants that, in combination, increase breast cancer risk significantly. Single Nucleotide Polymorphisms (SNPs) are the most common type of variant within a DNA sequence, with over 10 million SNPs estimated to occur within the human genome [21]. SNPs often explain normal variation between individuals and frequently have minimal known functional impact [22] However, when a SNP occurs within a gene or within the regulatory region near a gene, the gene's function can be affected, and this can contribute to disease development. Many of these SNPs have been singularly associated with a very small increase in BC risk. These BC-associated SNPs occur much more frequently in the population than the pathogenic variants found in high and moderate risk genes, but an individual SNP confers a much lower risk of BC development (Fig. 2). However, with over 300 such SNPs identified to date and the risks from each acting multiplicatively, ∼30% of familial heritability can be attributed to the known SNPs [23,24].

### 1.2 GWAS – Genome Wide Association Studies

Genome Wide Association Studies (GWAS) have genotyped between hundreds of thousands to millions of SNPs with the purpose of discovering variants that occur at a significantly higher frequency (usually with a *P*-value of <5 × 10^−7^) in disease-affected vs unaffected (control) individuals [26]. Using the frequencies of these variants, an odds ratio (OR) can be calculated which indicates the probability of an outcome based on an exposure; in this case, the development of breast cancer based on specific genetic variants [27]. GWAS have consistently linked certain SNPs within the genome to breast cancer development, having identified over 170 genomic regions that are associated with breast cancer risk and pinpointing over 190 likely target genes, with new studies and increasing sample sizes continually identifying new target genes and genomic loci associated with breast cancer risk [28,29]. Within these genomic regions, GWAS have identified over 300 SNPs associated with BC development. In addition to finding loci associated with breast cancer, these studies have also extended our understanding of the heritability of breast cancer with SNPs located in regulatory zones, many strongly enriched for transcription factor binding sites [30].

There is a need to expand GWAS studies to include more participants with substantial focus on detection of SNPs in subtype-specific variants (specifically ER-negative and Triple Negative (TN) tumours which cannot be prevented by endocrine therapies) as well as including individuals of diverse ethnicities beyond those of white European origin.

### 1.3 Polygenic risk scores

Polygenic Risk Scores (PRS) describe the combined result of numerous risk-inducing susceptibility variants, identified in GWAS [31]. PRS considerably improve risk prediction even though most of the susceptibility variants confer only a small risk individually. However, in conjunction, the multiplicative effect of the variants can dramatically influence risk. Generally, PRS are calculated by multiplying the published per-allele ORs for each risk SNP and these scores are used to improve the stratification of breast cancer risk on a population level [32,33]. It is vital that the mean PRS is calibrated to risk within the specific population being assessed.

The expected allele frequency (EAF) and OR for each SNP is used to calculate the risk score for each genotype using: *p*^*2*^ + *2pq* + *q*^*2*^ *= 1* where p is the risk allele and q is the non-risk allele.

*P*^*2*^ is the homozygous risk allele frequency, *2pq* is the heterozygous risk allele frequency *q*^*2*^ is the homozygous non-risk allele frequency.

The average population risk for the non-risk allele genotype is then calculated using the equation: (*p*^2^* published OR^*2*^*)* + (pq*published OR) + *(q*^2^*1)

The population adjusted risks for each of the three genotypes is calculated by:

*(published OR*^*2*^*/average population risk)* for two risk alleles *(published OR/average population risk)* for one risk allele *(1/average population risk)* for no risk alleles.

[34,35].

### 1.4 Discrimination and calibration

For a PRS to have clinical utility it is vital to have accurate discrimination and calibration. Discrimination is defined by correctly classifying whether an individual is either a case (BC affected) or control (BC non-affected). Calibration describes the agreement between observed and estimated incidences in the population [36,37].

Traditionally, many studies have used the discriminatory ability to assess the validity of a PRS. The area under the curve (AUC), defined as the probability that a randomly selected individual with a disease will be assigned a higher risk than a randomly selected individual without the disease, is the most used statistic for assessing discrimination. AUCs of 50% and 100% indicate that a model has no or perfect discrimination, respectively. Typically, PRS alone have modest discrimination at ∼0.60 however when considering PRS use in breast cancer, modest discrimination still has the potential to identify a large portion of the population at higher risk of disease development [38].

Calibration is typically assessed by calculating an observed and estimated odds ratio so that an O/E ratio of 1.0 demonstrates perfect calibration [39]. It is well known that calibration is often a problem when implementing PRS for breast cancer in populations outside of that which the PRS was designed in. Whilst the PRS often discriminates well between cases and controls, there is often a significant over or under prediction that needs to be corrected to accurately report an individual's risk [40,41].

### 1.5 Developing breast cancer PRS

Discovery of novel SNPs associated with breast cancer development in the larger GWAS, have resulted in expansion of the number of these SNPs incorporated in PRS to improve risk stratification. The history of the development of breast cancer PRS has been summarised in Table 2.

**Table 2:** Discrimination statistics for breast cancer PRS with increasing number of SNPs in populations of white European origin [3,33,[42], [43], [44], [45], [46], [47], [48], [49], [50], [51], [52], [53], [54]].

PRS	AUC	Cases/Controls	Year	Study Reference
SNP18	0.590	364/1605	2016	Evans
SNP22	0.650	1143/892	2012	Sawyer
SNP24	0.590	1496/2869	2017	Li
SNP32	0.580	6009/7827	2012	Hüsing
SNP77	0.620	33,673/33,381	2015	Mavaddat
SNP77	0.610	750/405	2016	Dite
SNP77	0.603	11,428/18,323	2019	Mavaddat
SNP83	0.590	387/387	2016	Shieh
SNP86	N/A	4291/19,968	2020	Hughes
SNP143	0.650	405/1668	2020	Brentnall
SNP313	0.630	11,428/18,323	2019	Mavaddat
SNP313	0.653	667/332	2022	Saloustros
**PRS beyond SNPs**	**AUC**	**Cases/Controls**	**Year**	**Study Reference**

SNP18 (+TC/MD)	0.670	466/8897	2018	Van Veen
SNP18 (+TC/DR)	0.634	525/1410	2022	Evans
SNP77 + (PD)	N/A	3628/5126	2019	Vachon
SNP143 (+TC/DR)	0.677	525/1410	2022	Evans
SNP143*	0.650	405/1668	2020	Brentnall
SNP313 (+TC/DR)	0.665	525/1410	2022	Evans
SNP5218 (LDpredict)	0.690	6586/157,895	2019	Khera

TC, Tyrer-Cuzick (classic risk factors); MD, Mammographic Density; DR, Density Residual (mammographic density); PD, Percent Density; LDpredict, Linkage Disequilibrium (predicted SNPs based on linkage disequilibrium). There are no reported discrimination statistics for SNP86. *AUC reported for PRS only.

Evans et al. conducted a case-control study where SNP18 was used to discover the role of this PRS in predicting risk of women who have no pathogenic variant in either of the *BRCA1/2* genes [42]. SNP18 was found to be predictive of BC (Interquartile range (IQR) OR 1.55, 95% CI 1.29 to 1.87, O/E 96%). There is currently an unmet clinical need to identify women who are at increased BC risk but without such high risk gene variants this study demonstrated that a PRS can help to identify these women. The same group also showed in a cohort of women recruited from the UK screening population that SNP18 PRS was higher in case patients (median, 1.12; IQR, 0.87–1.33) than controls (median, 1.01; IQR, 0.77–1.19), with near perfect calibration (unadjusted O/E OR, 1.03; 95% CI, 0.74–1.32) [53].

Beyond SNP18, SNP77 was one of the next well-validated breast cancer PRS’. In a study of ∼33,000 breast cancer cases and ∼33,000 controls of European origin, SNP77 was shown to stratify breast cancer risk in women with family history as well as those without [46]. Lifetime breast cancer risk was stratified as 8.6% and 24.4% in the lowest and highest quintiles respectively, for women with a first-degree relative with a history of breast cancer and 5.2% and 16.6% respectively, for women with no family history. Mavaddat et al. showed that using this PRS, women in the highest 1% were at 3-fold increased risk of breast cancer development when compared with women in the middle quintile. Other studies have confirmed similar ORs with the 77-SNP PRS (OR = 1.52 per SD, 95% CI 1.45–1.59) [54] and suggested that it explains ∼12.6% of the familial relative risk of breast cancer [55].

As more BC associated SNPs were identified a panel of 94 SNPs were examined with the best discriminatory accuracy coming from 86 of these 94 SNPs. These 86 SNPs thus made the SNP86 PRS [52]. This PRS was reported to be highly predictive of overall breast cancer status with P values of P = 6.4 × 10^−66^ and P < 10^−325^ in the two validation cohorts used. Odds ratios per SD were also highly consistent between validation sets (OR = 1.45, 95% CI 1.39–1.52 and OR = 1.47, 95% CI 1.45–1.49).

SNP143 is the next largest well-validated PRS for breast cancer risk and was described by Brentnall et al. when they developed this PRS from a panel of 172 SNPs linked to breast cancer by GWAS [49]. This study found that SNP143 was well calibrated with an O/E ratio of 1.10 (95% CI 0.86–1.34). SNP143 has been further validated by Evans et al., 2022 in combination with mammographic density and a gene panel (Table 2) [56].

SNP313, developed from studies conducted by the Breast Cancer Association Consortium, is predictive of breast cancer risk with an OR of 1.61 (95% CI 1.57–1.65) and an AUC of 0.630 (95% CI 0.628–0.651), demonstrating a good discriminatory ability in white European populations [33].

### 1.6 PRS with other risk factors

In breast cancer risk prediction models, the use of other known risk factors in combination with PRS has been shown to enhance prediction (Table 2). Well-validated PRS are used in joint association with mammographic density among other classic risk factors to improve risk stratification. Van Veen et al. demonstrated that SNP18 combined with risk data from the Tyrer-Cuzick model and mammographic density identified 16% of cases and 9.5% of controls that moved into the increased risk category (>5% 10-year risk) compared with using only the Tyrer-Cuzick model. They also found that 5% of cases and 4% of controls moved out of the high-risk category demonstrating the ability of PRS to not just place someone at higher risk but also to stratify those women who are at medium or low risk [53]. Van Veen et al. also found that the multiplicative effect of incorporating risk factors improved the models’ discriminatory ability with AUC increasing from 0.58 with Tyrer-Cuzick alone, to 0.64 with the addition of mammographic density to a high of 0.67 when the SNP18 PRS was included as well.

It has been shown that total risk stratification is improved for mammographic density when a SNP77 PRS is also taken into consideration, as shown by Vachon et al. [54] This study found that when considering mammographic percent density (PD), OR fell slightly from 1.45 (95% CI 1.38–1.52) to 1.42 (95% CI 1.36–1.50) when PD was adjusted for the SNP77 PRS.

Brentnall et al. showed that SNP143 used in combination with mammographic density and classic risk factors improved risk stratification [49]. Adding mammographic density and the SNP143 PRS to the Tyrer-Cuzick model classic risk factors, increased the percentage of the population in the lowest (<1.4% 10-year risk) and highest (8% + 10-year risk) risk categories. For cases, using Tyrer-Cuzick alone, 1.2% were in the lowest and 4.2% were in the highest risk groups. After including the SNP143 PRS and mammographic density, this increased to 9.4% in the lowest and 14.6% in the highest risk groups respectively. 64.4% of cases were placed in the low-moderate risk group (1.4–3.5% 10-year risk) using Tyrer-Cuzick alone with this decreasing to 33.1% using the combination of all available risk factors. The percentage decrease in the low-moderate risk group demonstrates that individuals are more appropriately stratified and placed in the lowest and highest risk groups when the PRS is used in combination with classic risk factors and mammographic density. For controls, using only the Tyrer-Cuzick model places 1.2% at the lowest risk and 2.2% at the highest risk whereas this increases to 22.3% in the lowest risk group and 7.4% in the highest risk group after inclusion of mammographic density and the SNP143 PRS. Again, this demonstrates the ability of PRS beyond classic risk factors to place an individual at low risk as well as identifying those at higher risk, much improving risk stratification across the whole population.

A comprehensive study by Evans et al., in 2022 showed the incremental effects of adding mammographic density and different PRS’ comprising increasing number of SNPs on risk stratification in a cohort of ∼500 cases and ∼1500 controls [3]. This study used SNP18, SNP143 and SNP313 to assess the role of PRS using increasing numbers of SNPs in the same population and how risk stratification changes with each PRS. The study showed an incremental improvement in stratification with increasing numbers of SNPs with the PRS being a much greater factor in risk discrimination than pathogenic variants in moderate or high-risk genes. The best performing combination gave an AUC of 0.684 (0.652–0.715). Overall, it was possible to identify 20.9% of controls and 42.5% of breast cancer cases at actionable NICE defined moderate-(10year risk 5–7.99%) or high (≥8%) risk categories.

### 1.7 PRS and moderate/high risk gene pathogenic variants

PRS have been shown to stratify risk beyond the ability of single high-risk gene pathogenic variants. Kuchenbacker et al. demonstrated large changes in the absolute risk of breast cancer development in a cohort of women (7797 *BRCA1* cases; 4330 *BRCA2* cases) from the Consortium of Investigators of Modifiers of *BRCA1/2* (CIMBA) with the use of a PRS [57]. One study found that SNP86 modifies the risk of women who are carriers of high and moderate penetrance gene variants for breast cancer. Gallagher et al. found that whilst risk stratification was better for non-carriers and women with a *CHEK2* pathogenic variant (OR = 1.47, 95% CI 1.45–1.49; OR = 1.49, 95% CI 1.36–1.64, respectively), SNP86 was still able to modify risk prediction in carriers of *ATM*, *PALB2*, *BRCA1* and *BRCA2* with odds ratios ranging from 1.20 for *BRCA1* to 1.37 in *ATM* [58].

Another study examining 26,798 cases and 26,127 controls from the Cancer Risk Estimates Related to Susceptibility consortium evaluated each participant for pathogenic variants in various high and moderate risk genes including *BRCA1, BRCA2, ATM, CHEK2, PALB2, BARD1, BRIP1, CDH1* and *NF2.* [59] In this study, Gao et al. found that PRS’ may be able to reclassify more than 30% of *CHEK2* and around 50% of *ATM* pathogenic variant carriers as having a lower-than-expected lifetime breast cancer risk (<20%). On a clinical level, this may provide significant reassurance and help to plan the most appropriate screening and preventive measures.

### 1.8 PRS and age

Most genetic studies linking risk prediction to breast cancer focus on generalised breast cancer risk with no specified age group. A large portion of breast cancer occurs in women aged >50 years, however, women affected by breast cancer at younger ages stand to have a greater number of life-years saved with successful early detection and treatment than their older counterparts. One study estimated that there is a 77% 10-year survival if diagnosed aged <40 years vs an 87% 10-year survival if diagnosed >50 years) [60]. Young-onset breast cancers often are phenotypically more aggressive and have some differences in aetiological factors to breast cancers seen in older women. In a study of 185 premenopausal women with invasive breast cancer at the European Institute of Oncology, the youngest women, who were less than 35 years old, had a higher proportion of triple negative cancers (P < 0.001), a higher percentage of cancers with lymphovascular invasion (48.6% vs 37.3%, P = 0.006) and a higher proportion of higher-grade tumours (P < 0.0001) compared with the cohort of women between the ages of 35–50 [61].

There is a need to expand GWAS to include more young women and potentially identify SNPs that are linked to an early-onset diagnosis of breast cancer. There is also the need to enrich for ER-negative SNPs in a PRS used in a young population due to the higher proportion of triple negative cancers seen in this cohort.

### 1.9 PRS and subtype specificity

To improve the ability to construct a subtype-specific PRS, there is a need to carry out GWAS in populations of patients that develop ER-negative and TN tumours. Alternatively, to increase the power for identification of ER-negative variants, association results for *BRCA1* pathogenic variant carriers (*BRCA1* predisposes predominantly to ER-negative tumours in younger women) within databases such as CIMBA, as well as BCAC ER-negative association results could be combined to perform a meta-analysis which enriches the ability for GWAS to discover ER-negative SNPs. Until then, research has shown some evidence of breast cancer PRS having some subtype specific predictive ability.

The SNP143 PRS has been shown to be a risk factor for both ER+ and ER-negative breast cancers [49]. Using participants from the CIMBA study, Kuchenbaecker et al. found that the PRS for ER-negative breast cancer showed the strongest association with breast cancer risk in *BRCA1* carriers (per SD hazard ratio = 1.27, 9%% CI 1.23–1.31, p = 8.2 × 10^−53^) with reduced but still significant associations with ER-positive and overall breast cancers in both *BRCA1* and *BRCA2* carriers [57].

The most comprehensive study investigating the use of PRS for prediction of specific breast cancer subtypes was carried out by Mavaddat et al., in 2019 and used both the SNP77 and SNP313 PRS [33]. In a sample of 11,428 cases and 18,323 controls, they found that the SNP77 PRS and SNP313 were both predictive for ER-positive and ER-negative breast cancer in addition to overall BC (Table 3). SNP313 provided better discrimination than SNP77 as expected with higher AUCs for overall BC in both the prospective test set and validation cohort. However, when considering subtype-specific disease, in both the 77-SNP and 313-SNP PRS, the odds ratios for ER-positive breast cancer, and overall breast cancer, were higher than for the ER-negative cancers.

**Table 3:** Reproduced from Mavaddat et al., 2019 [33].

		Prospective test set			Validation set		
		OR	95% CI	AUC	OR	95% CI	AUC
SNP77 PRS	Overall BC	1.46	1.42–1.49	0.603	1.49	1.44–1.56	0.612
	ER-positive	1.52	1.48–1.56	0.615	1.56	1.49–1.63	0.623
	ER-negative	1.35	1.27–1.43	0.584	1.40	1.30–1.50	0.596
SNP313 PRS	Overall BC	1.61	1.57–1.65	0.630	1.65	1.59–1.72	0.639
	ER-positive	1.68	1.63–1.73	0.641	1.74	1.66–1.82	0.651
	ER-negative	1.45	1.37–1.53	0.601	1.47	1.37–1.58	0.611

ER-negative risk prediction was still attenuated when compared to ER-positive breast cancer risk, most likely reflecting the fact that ER-negative disease is less common than ER-positive disease with correspondingly fewer cases in GWAS. However, a study by Michailidou et al. estimated the heritability of ER-negative disease is like that of overall breast cancer, indicating that increasing sample sizes should improve the power of ER-negative PRS [30,62].

Mavaddat et al. further assessed the associations between SNP313 PRS and breast cancer risk by first-degree family history of breast cancer. They found a smaller ER-positive PRS OR (0.91, p = 0.004) for women with a family history, indicating that PRS ORs for this subtype are higher in women without a family history (OR = 1.71 (95% CI 1.65–1.78)) than with a family history (OR = 1.55 (95% CI 1.48–1.65)). When they compared ER-negative breast cancers, there was also a higher OR for women without a family history (OR = 1.45 (95% CI 1.36–1.57)) than with a family history (OR = 1.40 (95% CI 1.27–1.55)) however the disparity was not as large as in ER-positive breast cancers [33].

### 1.10 PRS and ethnicity

A major challenge within the formulation and use of PRS is ensuring that they are equally suitable across patients of all ethnicities. Should PRS not be appropriately recalculated, their use in these populations is limited, further exacerbating existing ethnic disparities within healthcare systems [63]. This is an obstacle for people of ethnic minorities across the world, whether in a higher income country with a more well-established healthcare system in which they are inadequately represented or in a lower income country where healthcare and research are often limited due to financial constraints.

To date most BC variants have been discovered in GWAS’ of white European populations. However, many of these genetic risks are not transferrable to other populations, with some variants causing risk in one population and being protective in another. One study found that out of approximately 100 variants identified to increase risk in European and Asian individuals, 30–40% were protective in a population of African heritage [64]. These studies provide evidence that GWAS studies should be carried out in a population specific manner, particularly in non-European individuals, with all heritages considered as risk stratification is not automatically transferable from one population to another.

Evans et al. carried out a retrospective case-control study where they evaluated the role of the SNP18 and SNP143 PRS’ in populations of ethnic minorities having been historically well-validated in white European populations [56]. They found that both PRS overestimated risk of breast cancer in all ethnicities with the overestimation being highest in women of African heritage. When combining women of all ethnicities (black, Asian, mixed, and Jewish) into one non-white group, SNP143 was estimated to cause a potential mean 40% overestimation in breast cancer risk. For SNP143, they estimated a breast cancer overprediction for controls of 26% in Jewish, 91% in black, 29% in Asian and 15% in mixed ethnicity populations.

Some commercially available PRS include women of Ashkenazi Jewish ancestry in an ethnic group with white European women, although we have shown that risk is overpredicted in this group. In a study examining an Ashkenazi group from Manchester with a larger validation cohort from Israel, the Ashkenazi population from Manchester showed ∼20% overprediction for breast cancer risk when using SNP142. When the per-allele odds ratios were adjusted using published Ashkenazi Jewish effect allele frequencies and published odds ratios, risk prediction was successfully recalibrated with controls’ mean PRS correcting back to unity (unpublished data).

GWAS research in non-white Europeans needs to be improved with one 2019 study demonstrating that >78% of participants in published GWAS are of white European descent with 71.8% of this population being from only three countries, namely the United States, the United Kingdom and Iceland [65,66]. Whilst there has been some improvement since 2009, when an analysis documented that 96% of GWAS participants were of white European descent, there is still a need to widen participation in GWAS. By 2016, the proportion of non-white European individuals with published GWAS data had risen to nearly 20%, however most of this increase is attributable to Asian populations with representation for African, Latin American, Hispanic, and indigenous populations still very low.

When checking the GWAS diversity monitor, launched in *Nature Genetics* in 2020, with the purpose to track GWAS participants in real time, by August 2022 over 95% of participants in all known GWAS were white European, demonstrating the continuing lack of diversity and unmet clinical need to perform GWAS in non-white European populations. Of the remaining ∼4%, only 0.30% are of African descent, 0.25% Hispanic/Latin American, 0.65% mixed with the extra ∼3% constituting Asian participants [67].

It is vital that an individual's PRS is compared to population specific distribution, often done using Principal Component Analysis, to ensure that the right adjustments can be made depending on which ethnicity the individual has [68], as in most studies, ethnicity is self-declared. With increasing intermixing across populations of all ancestries, one approach may be to develop an assay which uses genetic markers to determine an individual's ethnicity. Integrating ethnicity associated SNPs into breast cancer PRS design may be a potential solution to this developing issue.

### 1.11 Are PRS suitable for clinical use?

Research studies continue to demonstrate the capabilities of PRS for breast cancer risk prediction, but PRS have not yet been implemented fully in any clinical setting [69,70]. Whilst PRS have mild-moderate discrimination (lower AUCs), this would be expected for a single risk factor. PRS become clinically useful when used as an addition to existing risk models which consider clinical and lifestyle risk factors, and mammographic density as evidenced previously [71].

However, to implement PRS at a clinical setting, there must be an understanding as to how a PRS would affect a patient on an individual level. Should a breast cancer PRS stratify a woman at higher risk of breast cancer development, this could lead them to make informed decisions regarding preventative measures such as taking medication, undergoing bilateral mastectomies and/or lifestyle modifications [72]. Nevertheless, there is a difference between relative risk and translating this into absolute risk that facilitates clearer understanding for an individual. For example, if a woman has a 16% chance of developing breast cancer in her lifetime compared with an 11% population risk, that patient has nearly 50% increased risk of developing breast cancer although in absolute terms this is only a 5% increased risk from population level. It is vital that patients are not needlessly exposed to anxiety regarding their genetic risk nor unnecessary radiation via increased mammographic screening, which is why PRS must be appropriately used by a clinician/healthcare worker and effectively communicated to their patient.

As PRS are relatively novel in breast cancer risk prediction, it is yet to be determined about the best approach to communicate a personalised risk score to a patient and if patients want to know this information compared with standard screening. PROCAS (Predicting the Risk of Cancer at Screening), WISDOM (Women Informed to Screen Depending on Measures of Risk) and MyPeBS (My Personalised Breast Screening) are all large-scale studies that investigate the viability of breast cancer PRS’ and how their clinical implementation could be facilitated [[73], [74], [75]].

## 2 Summary

Breast cancer PRS’ remain a keystone in personalised risk prediction medicine and have been shown to refine a patient's risk beyond classic risk factors. PRS have the potential to prevent many cancer deaths via early detection and risk-reducing measures. However, further work needs to be done to improve the use of PRS in women of non-white European ancestry as currently these groups are having their risk significantly overpredicted. To implement PRS on a clinical scale, research must devise how to effectively communicate this risk on an individual scale.

## Ethical approval

Ethical approval not required.

## Funding

DGE and SJH are supported by the Manchester National Institute for Health Research (NIHR) Biomedical Research Centre (IS-BRC-1215-20007). ER is funded by Cancer Research UK ACED International Alliance for Cancer Early Detection C19941/A27859.

## Declaration of competing interest

No authors have conflicts of interest to disclose.
